# Development and implementation of the AIDA International Registry for patients with Behçet’s disease

**DOI:** 10.1007/s11739-022-03038-1

**Published:** 2022-07-14

**Authors:** Antonio Vitale, Francesca Della Casa, Gaafar Ragab, Ibrahim A. Almaghlouth, Giuseppe Lopalco, Rosa Maria Pereira, Silvana Guerriero, Marcello Govoni, Petros P. Sfikakis, Roberto Giacomelli, Francesco Ciccia, Sara Monti, Piero Ruscitti, Matteo Piga, Claudia Lomater, Abdurrahman Tufan, Daniela Opris-Belinski, Giacomo Emmi, José Hernández-Rodríguez, Ali Şahin, Gian Domenico Sebastiani, Elena Bartoloni, Nurullah Akkoç, Özgül Soysal Gündüz, Marco Cattalini, Giovanni Conti, Gulen Hatemi, Armin Maier, Paola Parronchi, Emanuela Del Giudice, Sukran Erten, Antonella Insalaco, Francesca Li Gobbi, Maria Cristina Maggio, Farhad Shahram, Valeria Caggiano, Mohamed Tharwat Hegazy, Kazi Nur Asfina, Maria Morrone, Leandro L. Prado, Rosanna Dammacco, Francesca Ruffilli, Aikaterini Arida, Luca Navarini, Ilenia Pantano, Lorenzo Cavagna, Alessandro Conforti, Alberto Cauli, Elena Maria Marucco, Hamit Kucuk, Ruxandra Ionescu, Irene Mattioli, Gerard Espinosa, Olga Araújo, Burak Karkaş, Claudia Canofari, Jurgen Sota, Ahmed Hatem Laymouna, Asma. A. Bedaiwi, Sergio Colella, Henrique Ayres M. Giardini, Valeria Albano, Andrea Lo Monaco, George E. Fragoulis, Riza Can Kardas, Virginia Berlengiero, Mohamed A. Hussein, Francesca Ricci, Francesco La Torre, Donato Rigante, Ewa Więsik-Szewczyk, Micol Frassi, Stefano Gentileschi, Gian Marco Tosi, Marilia Ambiel Dagostin, Ayman Abdel-Monem Ahmed Mahmoud, Maria Tarsia, Giovanni Alessio, Rolando Cimaz, Teresa Giani, Carla Gaggiano, Florenzo Iannone, Paola Cipriani, Mariam Mourabi, Veronica Spedicato, Sara Barneschi, Emma Aragona, Alberto Balistreri, Bruno Frediani, Claudia Fabiani, Luca Cantarini, Autoinflammatory Diseases Alliance (AIDA) Network

**Affiliations:** 1 Rheumatology Unit, Policlinico “Le Scotte”, Department of Medical Sciences, Surgery and Neurosciences, Research Center of Systemic Autoinflammatory Diseases and Behçet’s Disease Clinic, University of Siena, viale Bracci 16, 53100 Siena, Italy; 2 Section of Clinical Immunology, Department of Translational Medical Sciences, University of Naples Federico II, Naples, Italy; 3 Rheumatology and Clinical Immunology Unit, Internal Medicine Department, Faculty of Medicine, Cairo University, Giza, Egypt; 4 Faculty of Medicine, Newgiza University (NGU), Giza, Egypt; 5 Rheumatology Unit, Department of Medicine, College of Medicine, King Saud University, Riyadh, Saudi Arabia; 6 College of Medicine Research Center, College of Medicine, King Saud University, Riyadh, Saudi Arabia; 7 Rheumatology Unit, Department of Emergency and Organ Transplantation, University of Bari, Bari, Italy; 8 Rheumatology Division, Faculdade de Medicina, Hospital das Clinicas (HCFMUSP), Universidade de Sao Paulo, Sao Paulo, Brazil; 9 Department of Ophthalmology and Otolaryngology, University of Bari, Bari, Italy; 10 Rheumatology Unit, Department of Medical Sciences, Azienda Ospedaliero-Universitaria S. Anna-Ferrara, University of Ferrara, Ferrara, Italy; 11 Joint Academic Rheumatology Program, 1st Department of Propedeutic Internal Medicine, School of Medicine, National and Kapodistrian University of Athens, Athens, Greece; 12 Rheumatology, Immunology and Clinical Medicine Unit, Department of Medicine, Università Campus Bio-Medico di Roma, Rome, Italy; 13 Department of Precision Medicine, Università Degli Studi Della Campania Luigi Vanvitelli, Naples, Italy; 14 Rheumatology Department, IRCCS Policlinico S. Matteo Fondazione, University of Pavia, Pavia, Italy; 15 Rheumatology Unit, Department of Biotechnological and Applied Clinical Sciences, University of L’Aquila, L’Aquila, Italy; 16 Rheumatology Unit, Department of Medical Sciences, University and AOU of Cagliari, Cagliari, Italy; 17 AO Mauriziano, Universita degli Studi di Torino, Academic Rheumatology Centre, Turin, Italy; 18 Division of Rheumatology, Department of Internal Medicine, Faculty of Medicine, Gazi University, Ankara, Turkey; 19 Carol Davila University of Medicine and Pharmacy, Bucharest, Romania; 20 Department of Experimental and Clinical Medicine, University of Florence, Florence, Italy; 21 Vasculitis Research Unit and Autoinflammatory Diseases Clinical Unit, Department of Autoimmune Diseases, Hospital Clinic of Barcelona, IDIBAPS, University of Barcelona, Barcelona, Spain; 22 Division of Rheumatology, Department of Internal Medicine, Sivas Cumhuriyet University Medical Faculty, Sivas, Turkey; 23 U.O.C. Reumatologia, Ospedale San Camillo-Forlanini, Rome, Italy; 24 Rheumatology Unit, Department of Medicine, University of Perugia, Perugia, Italy; 25 Division of Rheumatology, Department of Internal Medicine, School of Medicine, Manisa Celal Bayar University, Manisa, Turkey; 26 Pediatric Clinic, University of Brescia and Spedali Civili di Brescia, Brescia, Italy; 27 Pediatric Nephrology Unit, AOU Policlinic “G Martino”, University of Messina, Messina, Italy; 28 Division of Rheumatology, Department of Internal Medicine and Behçet’s Disease Research Center, Istanbul University-Cerrahpasa, Istanbul, Turkey; 29 Rheumatology Unit, Department of Medicine, Central Hospital of Bolzano, Bolzano, Italy; 30 Department of Maternal Infantile and Urological Sciences, Sapienza University of Rome, Polo Pontino, Rome, Italy; 31 Department of Rheumatology, Ankara City Hospital, Ankara, Turkey; 32 Division of Rheumatology, Ospedale Pediatrico Bambino Gesù, IRCCS (ERN-RITA Center), Rome, Italy; 33 Rheumatology Unit, San Giovanni di Dio Hospital, Florence, Italy; 34 University Department Pro.Sa.M.I. “G. D’Alessandro”, University of Palermo, Palermo, Italy; 35 Behcet’s Disease Unit, Rheumatology Research Center, Shariati Hospital, Tehran University of Medical Sciences, Tehran, Iran; 36 Department of Pediatrics, Ospedale “Giovanni XXIII”, Pediatric Rheumatology Center, AOU Consorziale Policlinico, Bari, Italy; 37 Department of Life Sciences and Global Health, Fondazione Policlinico Universitario A. Gemelli IRCCS, Rome, Italy; 38 Rare Diseases and Periodic Fevers Research Centre, Università Cattolica del Sacro Cuore, Rome, Italy; 39 Department of Internal Medicine, Pulmonology, Allergy and Clinical Immunology, Central Clinical Hospital of the Ministry of National Defence, Military Institute of Medicine, Warsaw, Poland; 40 Rheumatology and Clinical Immunology, Spedali Civili and Department of Clinical and Experimental Sciences, University of Brescia, Brescia, Italy; 41 Unit of Rheumatology, Azienda Ospedaliero-Universitaria Senese, Siena, Italy; 42 Ophthalmology Unit, Department of Medicine, Surgery and Neurosciences, University of Siena, Siena, Italy; 43 ASST G. Pini-CTO, Department of Clinical Sciences and Community Health, Research Center for Adult and Pediatric Rheumatic Diseases, University of Milan, Milan, Italy; 44 Division of Gastroenterology, Ospedali Riuniti Villa Sofia-Vincenzo Cervello, Palermo, Italy; 45 Bioengineering and Biomedical Data Science Lab, Department of Medical Biotechnologies, University of Siena, Siena, Italy

**Keywords:** International registry, Autoinflammatory diseases, Precision medicine, Rare diseases, Behçet’s disease, Uveitis

## Abstract

Purpose of the present paper is to point out the design, development and deployment of the AutoInflammatory Disease Alliance (AIDA) International Registry dedicated to pediatric and adult patients with Behçet’s disease (BD). The Registry is a clinical physician-driven non-population- and electronic-based instrument implemented for the retrospective and prospective collection of real-life data about demographics, clinical, therapeutic, laboratory, instrumental and socioeconomic information from BD patients; the Registry is based on the Research Electronic Data Capture (REDCap) tool, which is thought to collect standardised information for clinical real-life research, and has been realised to change over time according to future scientific acquisitions and potentially communicate with other existing and future Registries dedicated to BD. Starting from January 31st, 2021, to February 7th, 2022, 110 centres from 23 countries in 4 continents have been involved. Fifty-four of these have already obtained the approval from their local Ethics Committees. Currently, the platform counts 290 users (111 Principal Investigators, 175 Site Investigators, 2 Lead Investigators, and 2 data managers). The Registry collects baseline and follow-up data using 5993 fields organised into 16 instruments, including patient’s demographics, history, clinical manifestations and symptoms, trigger/risk factors, therapies and healthcare access. The development of the AIDA International Registry for BD patients will facilitate the collection of standardised data leading to real-world evidence, enabling international multicentre collaborative research through data sharing, international consultation, dissemination of knowledge, inclusion of patients and families, and ultimately optimisation of scientific efforts and implementation of standardised care.

*Trial registration* NCT05200715 in 21/01/2022.

## Introduction

In the field of rare diseases, evidence is often lower than that required since data are gathered mainly from case reports, case series or small observational studies. This makes difficult to taper clinical management of patients with rare diseases on a thorough research capable of underlying the course, manifestations, long-term outcomes and proper treatments in detail [1, 2]. With regard to systemic monogenic and multifactorial autoinflammatory diseases, this limitation has made urgent the creation of international registries capable of overcoming the research issues related to the limited number of patients referring to each single centre. Actually, the reduced epidemiological burden of rare diseases includes the poor availability of patients for recruitment into clinical trials, the lack of standardised care, and the lack of knowledge about the possible clinical presentation and natural history of these diseases, with consequent delay in diagnosis and absence of standardised treatment protocols. For these reasons, the AutoInflammatory Disease Alliance (AIDA) network has been launched with the aim to gather a worldwide group of physicians and researchers interested in sharing knowledge, experience, information and different perceptions on the clinical, therapeutic and research approach about autoinflammatory diseases. Focus of the AIDA Project is the development and maintenance of international registries for patients with different autoinflammatory diseases including Behçet’s disease (BD), monogenic autoinflammatory diseases, Still’s disease, Schnitzler’s syndrome, Periodic Fever, Aphthous stomatitis, Pharyngitis and cervical Adenitis (or PFAPA) syndrome, non-infectious uveitis, non-infectious scleritis, vacuoles, E1 enzyme/X-linked autoinflammatory somatic (or VEXAS) syndrome and undifferentiated autoinflammatory diseases. Each registry corresponds to a multicentre, non-interventional, observational cohort clinical study designed to share knowledge about autoinflammatory diseases and expand current evidence on such disorders through clinical research based on sufficiently large numbers of patients.

The purpose of the present paper is to point out the design, development and deployment of the AIDA International Registry specifically dedicated to patients with BD, explaining the rationale for this project, methods employed, and reporting on its implementation to date. Actually, although BD is relatively more frequent in the Countries along the Silk Road, it is a rare disease in most parts of the world [3–5]. Therefore, an International Registry specially tailored for this clinical entity represents an invaluable source of data about natural history and epidemiological, clinical and medical disease features, which may cover the shortcomings of traditional research.

## Materials and methods

### Study design

The AIDA Registry for BD patients is a clinical physician-driven non-population- and electronic-based registry meant to be as part of the AIDA project.

Data are collected both retrospectively, in reference to the information accrued at the time of the enrolment into the AIDA Registry, and prospectively with respect to clinical, therapeutic and socioeconomic information collected starting from the time of the inclusion into the Registry. Regarding the prospective collection, data have to be updated at least annually or in case of treatment change, as for posology adjustments and combination of different immunosuppressant agents.

According to its observational nature, the Registry captures real-world demographic, genetic, clinical, laboratory, and treatment details in a standardised format; long-term outcomes and prognostic variables are also collected when patients do not withdraw the consent to the project over time. Noteworthy, no specific variables or data entries exclusively related to the study protocol are required and only data derived from the standard routine management are collected. Any treatment approach administered prior or after the enrolment into the AIDA project and each posology change performed for the patients’ welfare may be described in the Registry; however, none of the prescribed therapies have been affected by the adherence to study in any way.

Participation is open to any centre managing BD patients regardless of location, medical specialty or type of practice setting. The only prerequisites to participate are obtaining approval from the local Ethics Committee and appointing a Principal Investigator who coordinates the study locally and Site Investigators responsible for the documentation and data entry for that site. There is neither cost nor financial compensation to participate, as data collected refer only to information routinely gathered in the clinical practice.

### Registry objectives

The primary objective of this BD Registry is to overcome the current fragmentation of clinical and research experience to carry out robust research based on a sufficiently large number of patients. The final goal is to obtain solid results with pioneering studies. The first steps will aim to better characterize the disease and response to treatment according to different discriminating factors including sex, age at onset, ethnic influence, geographical origin, body mass index, smoking habit, alcohol intake, disease duration, or diagnostic delay.

Other objectives include: (1) the search for symptoms clustering or the identification of any patients’ subset leading to potential prognostic or therapeutic implications; (2) the description of older and newly identified therapeutic solutions in terms of global efficacy and the specific role in different manifestations of the disease; (3) better understanding of the role of posology adjustments; (4) the description of socioeconomic impact of the disease in terms of national health system use (days of hospitalization, access to the emergency department, consult to the general practitioner) and evaluation of working capacity (presenteeism and/or absenteeism); (5) the behaviour of BD during pregnancy and postpartum period; (6) the impact of the disease (and treatments) on the cardiovascular risk; (7) the search for prognostic factors useful to personalise therapy according to short- and long-term prognosis; (8) the description of the best diagnostic pathway disregarding any influence by financial resources on health; (9) the change of natural history of the disease over time, to be eventually correlated with the development of new treatment approaches along with socio-economic and environmental changes; (10) the identification of demographic factors capable to influence BD onset, the severity of manifestations and response to treatments. Depending on the number of patients enrolled and according to future unmet needs, it will be possible to design specific and cutting-edge studies. Of note, it will be possible to easily identify patients to eventually include in future randomized controlled trials (RCTs), whose realization is difficult owing to the limited incidence and peculiar prevalence of the disease.

All the objectives of the Registry will be considered in the light of the machine learning data analysis, which represents a new way to statistically analyse clinical information and real-world data. This approach will pave the way for the optimisation of research and implementation of precision medicine.

### Inclusion/exclusion criteria

Inclusion criteria into the AIDA Registry for BD consist in the fulfilment of the International Criteria for Behçet’s disease (ICBD), International Study Group (ISG) criteria, and classification criteria for pediatric BD according to the age at disease onset [6–8]. Additionally, all patients enrolled have to provide their written informed consent after having been carefully informed about the project and its aims, long-term purposes, lack of any impact of the study on clinical practice, possibility to refuse entering the study without this affecting the clinical workflow at the reference centre, privacy-related security according to local and/or European regulations and the possibility of withdrawing from the study at any time. The parents or a legally acceptable representative and the adolescent subjects have to be willing and able to comply with the study requirements for the duration of the study. Otherwise, there are no exclusion criteria.

### Online data collection

Data are collected through Research Electronic Data Capture (REDCap), an electronic data capture tool developed at the Vanderbilt University Medical Center (VUMC) and hosted at Virginia Commonwealth University (Award Number UL1TR002649), which can be used also to develop patients’ registries. The software is distributed at no cost to members of the REDCap Consortium, which is a global collaboration of over 5,700 diverse institutions across 145 countries that have installed the software and collaborate to provide support [9].

Principal Investigators and Site Investigators included into the AIDA project are able to login the Registry through the REDCap web-interface, insert data on the instruments of the Registry and then review (and eventually complete) information provided by their own centre. None of the recruited Principal Investigators and Site Investigators are allowed to see information inserted by other Centres. The electronic data entry system of the Registry is in English.

While the public website (https://aidanetwork.org/en/) may be accessed by everyone who wants to learn more about the AIDA network, its objectives and how to participate to the project, the Registry website (https://sitbio.med.unisi.it/redcap/redcap_v12.2.1/index.php?pid=25) is hosted separate from the public website as a secure approach. Data entry is password-protected and collected data are stored on a secure server in the University of Siena, Siena, Italy.

### Ethics

The first national regulatory approval of the AIDA project has been obtained in June 2019 by the Ethics Committee of Azienda Ospedaliero-Universitaria Senese, Siena, Italy (Ref. N. 14951; NCT05200715). Later, expert centres for the diagnosis, clinical management and treatment of BD across Europe, the Middle East, Africa and America have been invited to approve the project and participate in the AIDA Registry.

Patients’ data are kept in accordance with the EU General Data Protection Regulations (GDPR) on the processing of personal data and the protection of privacy in the electronic communication (2016/679/EU) [10]. The Registry is run following the recommendations from the Declaration of Helsinki. Participation into the Registry is voluntary. After having received age-appropriate information sheets, patients enrolled (or their parents/caregivers) have to give their informed consent; minors aged ≥ 12 years are also required to provide their agreement before their inclusion in the study.

Both patients and Principal Investigators may withdraw their consent for the use of data for statistical analyses at any time. In case a patient withdraws the consent, no further data for that patient will be entered into the Registry and, if requested by the patient, all of his or her prior data will be deleted from the Registry soon after communication to the study promoter.

Patients do not receive any honoraria or other types of payment for participation in the registry, and no relationship to billing of the healthcare system or insurance companies have to be highlighted.

### Data quality management

In accordance with the recommendations for improving the quality of rare disease registries [11], a plan for ensuring data quality has been carefully implemented. In particular, inclusion and exclusion criteria have been explicitly defined; the investigators are required to enroll patients consecutively in the Registry, to minimise the risk of selection bias. In addition, the consistency and completeness of data entered will be periodically checked, along with the presence of coding errors; centers will receive specific queries in case of suspected discrepancies in the information collected or if missing data may affect the strength of the studies deriving from the Registry. A further important operation will be the control of duplicate cases, entered more times by the same Center or by different Centers to which a patient may have been referred.

An in-depth training of the Registry participants is also guaranteed through periodical audits, meetings, evaluations of the Registry growth modalities and protocol reviews.

### Statistical analysis

Statistical analysis will depend on the objectives to achieve from time to time on the basis of the data collected in the Registry; however, the statistical plan will include general principles related to descriptive statistics, correlations between groups and comparisons between subgroups. Statistical analysis will be performed also according to machine learning principles.

## Results

**Fig. 1:**
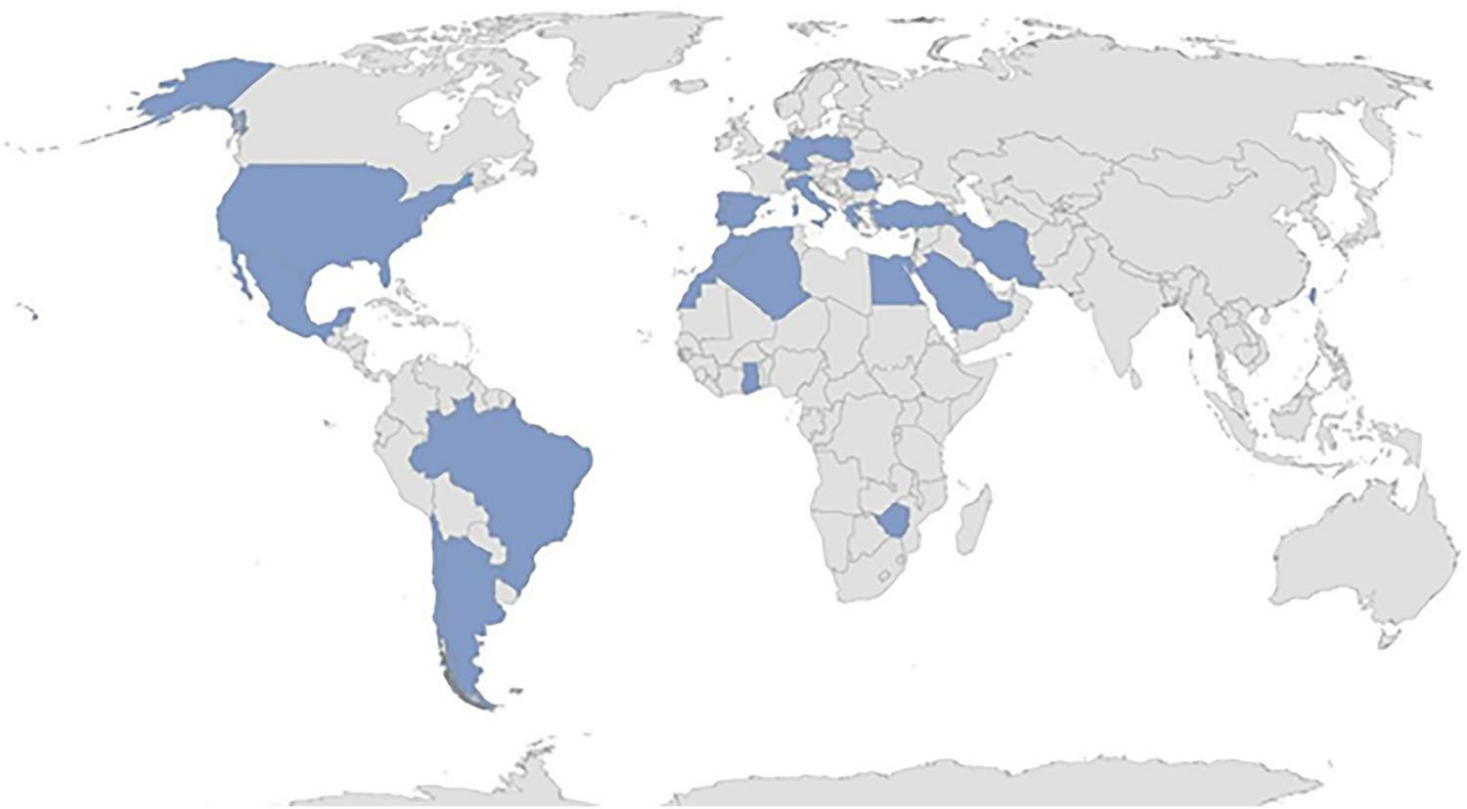
Worldwide distribution of the AIDA Network on February 7rd, 2022

The enrolment of patients with BD on the corresponding AIDA Registry has started on January 31st, 2021. The development and activation of this international Registry boasting the primary and essential purpose of obtaining solid scientific information from wide amount of real-world data is a result in itself. The project has quickly reached a wide geographic coverage capable of including patients from all over the world: in particular, 23 Countries (Algeria, Argentina, Belgium, Brazil, Chile, Egypt, Germany, Ghana, Greece, Iran, Italy, Lebanon, Mexico, Morocco, Poland, Portugal, Romania, Saudi Arabia, Spain, Taiwan, Turkey, United States, Zimbabwe) in 4 continents have been involved so far. As a whole, 110 centres around the world have joined the AIDA project and 35 (31,8%) have currently (February 7th, 2022) entered data on the Registry dedicated to patients with BD; 290 users (111 Principal Investigators, 175 Site Investigators, 2 Lead Investigators, 2 data managers) have applied for credentials to access the Registry. At current (February 7th, 2022), 636 patients (287 females/344 males; 5 missing values) have been enrolled. Figure 1 highlights the worldwide distribution of the AIDA network.

### Registry development

Clinical variables included into the study have been chosen to reconstruct the regular course of a complete patient's history and with the aim to respond to current unmet needs. As a whole, 5993 common data elements (fields) organised into 16 instruments (forms) constitute the Registry at current.

The full list of instruments and the time-points at which they should refer are listed in Table 1.

**Table 1:** List of instruments (to be meant as “forms”) included in the Registry dedicated to patients with Behçet’s disease, with the corresponding number of common data elements, time-points at which they should refer to and number of mandatory fields included

Instruments	Fields	Retrospective/prospective phase	N. of mandatory fields
Demographics	10	Retrospective phase	4
Consents	4	Retrospective/prospective phase	1
Diagnostic data and family history	35	Retrospective phase	2
Clinical features observed from the disease onset to the time of the enrolment	242	Retrospective phase	1
Ophthalmological assessment at the enrolment (or at the latest visit before enrolment)	380	Retrospective phase	0
Clinical diagnostic scores and criteria	9	Retrospective phase	0
Cardiovascular risk	24	Retrospective/prospective phase	2
Past and current treatments	1	Retrospective phase	0
Treatment with cDMARDs not associated with biologic agents—the retrospective phase	530	Retrospective phase	6
Treatment with small molecules not associated to biologic agents—the retrospective phase	931	Retrospective phase	12
Treatment with biologic agents—the retrospective phase	1606	Retrospective phase	14
Ophthalmological assessments performed over time, to be referred to the different treatment approaches attempted up to the time of enrolment	383	Retrospective/prospective phase	0
Fertility and pregnancy	14	Retrospective/prospective phase	3
Disease course and treatment during pregnancies	69	Retrospective/prospective phase	1
Follow-up visits—the prospective phase	1221	Prospective phase	51

*cDMARDs*: conventional disease modifying anti-rheumatic drugs

The common data elements correspond to an equal number of items that describe data on patient’s medical history regarding demographic, genetic and laboratory features, as well as symptoms at onset, symptoms developed over time, comorbidities, cardiovascular risk, work-up exams, pregnancies, past and current treatments, long-term clinical outcomes, and access to healthcare. The fields are organized in such a way as to appear only if patient’s clinical history makes necessary to answer them, thanks to a branching mechanism of questions. Therefore, only a part of the 5993 fields will appear to the investigators, and the number of questions to be answered in the Registry will depend exclusively on the complexity of the patient's clinical history. Conversely, the mandatory fields to necessarily answer consist in the date of birth, the living status, the date at the presentation of the first symptoms, the date at diagnosis, any ocular involvement (to eventually allow the opening of the forms dedicated to the ophthalmologic assessment), the date of the follow-up visits, the duration of therapies expressed in months. In the follow-up form, it will be mandatory to answer to whether the patient is treated with conventional disease modifying anti-rheumatic drugs (cDMARDs), biotechnological agents, small molecules; which cDMARD is eventually used, any reasons that induce the treatment discontinuation.

Data elements, where appropriate, are shared with other AIDA Registries dedicated to different autoinflammatory diseases; other bespoke data elements are specific of BD and have been added to meet the specific field of this disease in terms of clinical phenotype, clinimetry, possible complications, treatments not useful for patients with other autoinflammatory diseases, and the different follow-up approach planned for the prospective enrolment phase. In relation to this last point, longitudinal data are captured through a specific follow-up instrument.

Both the retrospective and the prospective instruments include the filling-in of the Behçet’s disease current activity form (BDCAF), which is a score used for the assessment of disease activity during the past 4 weeks, and the Behçet’s syndrome Overall Damage Index (BODI) score, developed to assess organ damage at a given time [12, 13]. In case of BD-associated ocular inflammatory involvement (in terms of uveitis, scleritis, macular edema, retinal vasculitis, and papillitis), the compilation of specific data drawn from ophthalmic examinations is also required. In particular, best corrected visual acuity, the classifications provided by the standardized uveitis nomenclature (SUN) working group, the grading scheme for vitreous haze according to Nussenblatt et al., the scleritis grading system, the standardised scoring system proposed by McCluskey et al. for patients with scleritis, the National Eye Institute Grading System for Vitreous Cells, the ASUWOG fluorescein angiography scoring system, and the ASUWOG indocyanine green angiographic scoring system are all part of the items required for BD patients suffering from ocular involvement [14–20].

Of note, this Registry has been designed to communicate with other existing or future registries with similar objectives. This is part of the sustainability of the project and represents a good practice to take into account when developing a new registry. Indeed, data often require to be merged from different registries with the purpose of answering old questions from different perspectives and facing new issues deriving from future unmet needs.

### Patients’ involvement

In recent years, patients have developed an increasing awareness of their role, which has become central in stimulating the research effort and quality of clinical management [21]. Actually, patient advocacy groups may help in many ways, by disseminating information, supporting the recruitment of patients, and taking part in regulatory processes. For these reasons, the Italian association of BD patients (S.I.M.B.A., *Associazione Italiana Sindrome e Malattia di Behçet*) has been actively involved into the AIDA project and other associations based in other countries will be invited to participate in a near future.

## Discussion

Networks are born to facilitate the spreading of knowledge and establish research collaboration between researchers, clinicians, industries, patient organizations, and single patients with their families. This is even truer in the field of rare diseases, as the small number of cases is a barrier to the translational research and makes the identification of a substantial cohort very difficult. In this context, the AIDA project and the corresponding Registry for patients with BD represent very useful tools to overcome the current fragmentation of knowledge and research regarding this rare disease. Actually, the management of BD requires the co-partnership of different specialties that must necessarily communicate with each other, including rheumatologists, ophthalmologists, geneticists, dermatologists, immunologists, gastroenterologists, phlebologists, internal medicine physicians, and paediatricians for patients with early-onset BD. As the AIDA Network has managed to include a large number of specialities, this project represents one landmark demonstration of how well a web-based worldwide collaboration may give benefit to the research and clinical management of this rare and complex disease. This collaboration may lead to a substantial increase of efforts from the pharmaceutical industry to develop new treatment approaches for BD.

To date, the AIDA project has fulfilled the expectations for the development of a platform dedicated to BD, where the scientific community may communicate, share experience, and actively participate in research efforts based on large-enough cohorts of patients to ensure previously undreamed projects. In this regard, the implementation and execution of a large International Registry may finally overcome the limitations of smaller individual studies [22], providing valuable and generalizable information on the disease. A large-scale, long-term, patient registry may provide additional evidence on the behaviour of BD in the short- and long-term, according to geographical distribution, different treatment approaches and many features capable to identify specific subgroups of patients. In this light, future research can aspire to a personalized medicine aimed at choosing the right treatment according to the baseline features, the risk to develop severe manifestations and complications, and on the basis of a specific organ involvement. Such a long-term observational Registry could allow an assessment of how the natural history of the disease is changing along with the varying therapeutic approaches and environmental conditions [23–25]. In addition, as the clinical manifestations of BD seem to change according to variable geographical conditions [26], an improvement of current diagnostic tools should be guaranteed on the basis of the protean BD spectrum observed in different countries. These objectives will be reached also thanks to the application of machine learning systems capable of deeply analysing the wide number of real-life data recruited.

The Registry may also provide an invaluable source of data to better understand the cardiovascular risk of BD patients and the behaviour of this disease in different periods of pregnancy (first, second, third trimester) and during the postpartum period. The prospective phase of the project will also provide more information on the socioeconomic impact of the disease and the benefits that national health systems could obtain from the treatments used. The Registry will also make it possible to assess the proper dosing and long-term benefit/risks of any treatment used for BD patients.

Its flexibility will allow improving the registry over time in accordance with new acquisitions derived from the international medical literature and in case of protocol amendments. Also, specific RCTs will be possible thanks to a potentially worldwide basis of enrolment, which will overcome issues related to the low incidence and prevalence of BD. In this regard, performing studies even on patients with rarer features of BD will be possible, as enrolment would be guided by a rapid search for patients presenting the characteristics required by a given study design.

While the first studies from the AIDA Registries have been published in recent times, several forthcoming papers are planned to specifically derive from real-world standardised data collected into the Registry dedicated to BD patients, especially in relationship with the description of baseline BD features, differences related to geographic distribution, description of current treatment approaches in different settings, and the way these treatments are effective in inducing a lower impact on a national health system. Deeper analyses and studies on some of the rarer subtypes of the disease are projected and will be realised according to the sample size reached in the coming years and according to the implementation of machine learning systems.

As a whole, real-world evidences deriving from this Registry will corroborate and improve current knowledge about BD. Indeed, although many data have been widely published during the last decades about the different aspects of the disease, they mainly derive from Countries with a high prevalence of the disease (countries along the ancient Silk route). This is an evidence for geographic discrepancies and clusters, factors affecting the disease course and outcomes, and response to treatment [27–30]. The present Registry gives the opportunity to assess collectively the different aspects of BD, with a specific focus on countries characterised by lower prevalence. In particular, differences in BD manifestations, onset and course along with the choice of treatment and management of patients should be highlighted for the first time. Assessing the distribution of patients worldwide will allow RCT capable of recruiting subjects from all over the world, thus minimising any bias related to geographical contexts.

In the next future, part of the efforts will be directed towards improving the sustainability of the project through the implementation of a clear development strategy, the definition of management mechanisms, and the regulation of the partnership and all the stakeholders involved. Moreover, collaboration with other projects and registries dedicated to BD will be sought, to maximise the results obtained from data collection in this AIDA Registry through different evaluations of the data according with other research perspectives.

A close collaboration will be also pursued with all the stakeholders interested with the AIDA Project, especially patient associations and professional organisations. In particular, a branch of the AIDA project defined as "AIDA for patients" is under development. “AIDA for patients” is an electronic system for collecting patient-reported data, thought with the aim of involving patients in the assessment of the disease status, the impact of BD on the quality of life and on socio-economic aspects; moreover, “AIDA for patients” was born to facilitate patients’ participation in the decision-making process both in terms of guidance of scientific research and in providing the collection of patients reported outcomes (PROs) to enhance a research based on real-life data.

All aspects of the Registry dedicated to BD patients will be subject of interim reports that will highlight the goals achieved in the future through meetings, congresses and dedicated papers.

The AIDA Registry for patients with BD has all the usual limitations of an observational research in general and registries, in particular. Entering data into the Registry requires time and attention, especially when patient's medical history is particularly complex, *i.e.,* characterised by multiple treatment approaches and many posology changes. Actually, while entering prospective data from follow-up visits takes a maximum of 10 min, entering retrospective data from a particularly complex BD patient can take many hours. In addition, the participating investigators are under no obligation to consecutively enrol all their BD patients, and unintended selection bias is possible. Nevertheless, beyond its limitations, this Registry has the potential and the geographical basis to really achieve all the objectives fixed. Moreover, we are currently developing “AIDA for patients” which will minimise missing values resolving, among other things, some of the quality limitations associated with data entry.

In conclusion, the development of the AIDA international Registry for patients with BD will facilitate the collection of standardised data enabling international multicentre collaborative researches through data sharing, implementation and optimisation of scientific efforts, international consultation, dissemination of knowledge, and inclusion of patients and families. Next steps will include the implementation of patients’ participation, new efforts to further disseminate the Registry by a broad international cooperative network, the inclusion of all the possible stakeholders, and the strengthening of the project sustainability.
